# Prevalence of Small-for-Gestational-Age and Its Mortality Risk Varies by Choice of Birth-Weight-for-Gestation Reference Population

**DOI:** 10.1371/journal.pone.0092074

**Published:** 2014-03-18

**Authors:** Joanne Katz, Lauren A. Wu, Luke C. Mullany, Christian L. Coles, Anne C. C. Lee, Naoko Kozuki, James M. Tielsch

**Affiliations:** 1 Department of International Health, Bloomberg School of Public Health, Johns Hopkins University, Baltimore, Maryland, United States of America; 2 Department of Newborn Medicine, Brigham and Women's Hospital, Boston, Massachusetts, United States of America; 3 Department of Global Health, George Washington University, School of Public Health and Health Services, Washington, DC, United States of America; National Institute of Child Health and Human Development, United States of America

## Abstract

**Background:**

We use data from rural Nepal and South India to compare the prevalence of small-for-gestational-age (SGA) and neonatal mortality risk associated with SGA using different birth-weight-for-gestation reference populations.

**Methods:**

We identified 46 reference populations in low-, middle-, and high-income countries, of which 26 met the inclusion criteria of being commonly cited and having numeric 10^th^ percentile cut points published. Those reference populations were then applied to populations from two community-based studies to determine SGA prevalence and its relative risk of neonatal mortality.

**Results:**

The prevalence of SGA ranged from 10.5% to 72.5% in Nepal, and 12.0% to 78.4% in India, depending on the reference population. Females had higher rates of SGA than males using reference populations that were not sex specific. SGA prevalence was lowest when using reference populations from low-income countries. Infants who were both preterm and SGA had much higher mortality risk than those who were term and appropriate-for-gestational-age. Risk ratios for those who are both preterm and SGA ranged from 7.34–17.98 in Nepal and 5.29–11.98 in India, depending on the reference population.

**Conclusions:**

These results demonstrate the value of a common birth-weight-for-gestation reference population that will facilitate comparisons of SGA prevalence and mortality risk across research studies.

## Introduction

Low birth weight (LBW) has long been recognized as an important risk factor for infant mortality, indirectly accounting for 60–80% of the 3.1 million neonatal deaths [1], [2]. LBW can occur due to prematurity or intrauterine growth restriction (IUGR), or both of these conditions. IUGR is defined as insufficient fetal growth and can be due to many factors including poor maternal nutrition, maternal infections, congenital defects, smoking, and placental conditions [3]. Small-for-gestational-age (SGA), which is commonly used as a measurable proxy for IUGR, is defined as birth weight below the 10th percentile of a reference distribution of weights specific to gestational ages, with some references providing sex-specific distributions. It was recently estimated that 32.5 million infants in low- and middle-income countries were born SGA, 19 million of whom were not LBW [4]. 53 percent (16.8 million) of these SGA infants were born in South Asia [4]. In low-income countries, a larger percent of LBW is due to IUGR than preterm [5]–[8]. SGA can arise from a genetic predisposition to small size or could be due to factors such as low maternal height, malnutrition, and/or infection during pregnancy. The genetic and constitutional contributions to SGA are generally felt to be small relative to these other factors, particularly in low- and middle-income contexts [9].

There are many different birth-weight-for-gestation reference populations, some of which have been used extensively to calculate the prevalence of SGA in the literature. In order to compare the prevalence of SGA across different studies, we wished to better understand what impact the choice of a reference population would have on SGA prevalence, and whether this choice would impact the estimate of mortality risk associated with being born SGA. To address this question, we conducted a literature review to identify all birth-weight-for-gestation reference populations that have been cited frequently. We then applied the 10^th^ percentile cut offs of each reference population to two community-based populations in rural southern Nepal and Tamil Nadu, India, and estimated the prevalence of SGA and its association with neonatal mortality using gestational age, birth weight, and mortality data.

## Methods

### Identification of reference populations

We identified an initial group of commonly used birth-weight-for-gestation reference population standards, solicited from experts (the Child Health Epidemiology Reference Group (CHERG) investigators) [10]–[16]. Through a snowball approach, we examined all reference populations cited in these papers, and examined the bibliographies for additional references. After four rounds, most of the references in each of these papers were recurrent. While this approach is likely biased towards the older English-language literature, we did identify several references in other languages (German, Dutch, French, Italian). We do not expect the potential bias to influence our findings significantly. The primary purpose in identifying different reference populations was to examine the extent to which the choice influenced the prevalence of SGA and the associated mortality risks. We categorized the reference populations by region: North America, South America, Europe, Asia, and Africa. If available, we recorded the years of data collection, setting or data source, number of live births, range of gestational ages included in the reference populations, method of gestational age assessment, socioeconomic status, maternal characteristics, and race or ethnicity of the population. Other key relevant characteristics included whether the growth curves were sex-specific, and whether stillbirths, multiple births, obstetric complications, or infants with congenital malformation were excluded. We also excluded reference populations where growth curves but not the actual 10^th^ percentile cutoffs were provided since we would have had to interpolate the 10^th^ percentile cut points imprecisely in these cases. We used Web of Science (Thompson Scientific, Inc.) and Google Scholar to identify the number of times the paper was cited. Only those cited more than ten times in either Web of Science or Google Scholar were used in the analysis, with the understanding that the publication may not necessarily have been cited as a reference population (Table S1 in File S1). However, because the original publications did specifically describe reference populations, we felt it likely that these were cited because they were reference populations.

### Datasets

We used data from two population-based randomized trials of neonatal interventions to calculate the prevalence of SGA using the reference populations described above. The Nepal Newborn Washing Study (NCT00109616) enrolled all infants born alive in 30 Village Development Committees of Sarlahi District in rural southern Nepal (at low altitude along the Indian border with Bihar) from September 2002 through January 2006 [17]. The trial tested the impact of a neonatal full body wipe with chlorhexidine or placebo at birth on neonatal mortality. The Vitamin A Study In Newborns (NCT00114868) enrolled pregnant women from two blocks of rural Tamil Nadu from June 1998 through March 2001 and randomized neonates to placebo or vitamin A supplementation within 48 hours of birth [18]. In both trials, study workers who were local village women identified pregnancies at around 4–5 months gestation, enrolled and interviewed the pregnant women, and followed them for pregnancy outcomes. Since most deliveries occurred at home, the village-based worker notified a high school graduate field worker when the birth occurred. The latter worker then went to the home to weigh the newborn with a digital infant scale, accurate to 2 g (Seca Digital Baby Scale Model 727, Columbia, Maryland). For deliveries that occurred in facilities, study workers waited until the mother returned home to weigh the infant. Facility birth weights were not collected or used in this analysis. In Nepal, gestational age was calculated by taking the mean of two estimates using the date of last menstrual period (LMP) by maternal recall, one obtained in mid-pregnancy and one at the birth of the live born infant. In India, gestational age was based on date of LMP by maternal recall at the time of pregnancy identification (around mid-pregnancy). Local event calendars were used to improve recall of dates. Gestational age was calculated in completed weeks rather than rounding to the nearest week.

The Nepali and Indian populations were similar with respect to many characteristics (Table 1). Facility deliveries were more common in South India (56% versus 8%), and maternal literacy was higher (46% versus 25%). The proportion of all live births weighed within 72 hours was higher in Nepal (84% versus 68%), perhaps reflecting more home births, and those weighed within 72 hours were weighed earlier in Nepal (median 16.5 hours) than in India (median 22.5 hours). Since birth weight was measured by study workers, those born in facilities were generally reached for a post-delivery visit and weighed only after the mother and infant returned home from the facility. This delayed the weighing of the infant relative to a home delivery. The prevalence of LBW was similar in both populations but prematurity was higher in Nepal (17% versus 13%). The overall neonatal mortality in each population was similar (around 30 per 1000 live births), but the mortality among those who survived long enough to be weighed was significantly lower in both studies, reflecting higher mortality risk among those not weighed. Infants who died before they were weighed were more likely to be preterm and have died soon after delivery. In Nepal, 6.9% of infants did not have a weight that could be used in this analysis (4.0% were weighed after 72 hours). In India, 23.7% of weights could not be used (17.1% were weighed after 72 hours). Gestational age was available on over 99% in both data sets. Further details of the studies are published elsewhere [17], [18].

**Table 1:** Characteristics of Mothers and Infants in the Population-based Trials in Nepal and India.

Characteristic	Nepal [17] a	India [18] b
Number live births	23,662	12,936
N (%) weighed within 72 hours	20,219 (85.4)	8,908 (68.9)
N (%) singletons with gestational age recorded and weighed within 72 hours	19,966 (84.4)	8,794 (68.0)
Median age at weighing (hrs)	16.5	22.5
Mean (SD) birth weight (g)	2,696 (422)	2,651 (399)
N (%) low birth weight (<2500 g)	6,441 (32.3)	4,944 (56.2)
Mean (SD) gestational age (wks)	39.3 (2.4)	39.5 (2.8)
N (%) preterm (<37 weeks)	3,463 (17.4)	1,170 (13.3)
Number of neonatal deaths (≤28 days)	325	159
Neonatal mortality per 1000 live births	16.3	18.1
% Maternal literacy	25.2	45.7
% Nulliparous	25.2	30.6
% Delivering in facility	8.0	56.1

a Missing Nepal of N = 19,966: gestational age 12, maternal literacy 10.

b Missing India of N = 8,794: gestational age 1.

### Data analysis

The overall and sex-specific prevalence of SGA in the Nepali and Indian trial populations was calculated by taking the number of infants whose weights fell below the 10^th^ percentile of each reference population for a specific gestational age. The range of gestational ages for which weight cutoffs was available varied widely by reference population (Table S1 in File S1). If the gestational age in the Nepali or Indian data fell outside the range for a particular reference population, the 10^th^ percentile weight cut point of the closest gestational age in the reference population was used. For example, if the reference population provided 10^th^ percentile cut points through 41 weeks gestation, that cutoff was used for a neonate with gestational age of 42 weeks or above. If sex-specific percentiles were available, the total prevalence of SGA was calculated using the sex-specific reference distributions.

To examine the association between SGA, preterm, and neonatal mortality (deaths within 28 days per 1000 singleton live births, weighed within 72 hours and with gestational age estimates), risk ratios (RR) and 95% confidence intervals were calculated for term-SGA, preterm-SGA, and preterm-appropriate-for-gestational-age (AGA), all with term-AGA as the reference. AGA is defined as birth weight being above the 10th percentile of the reference population at a specific gestational age. Forest plots of these RRs and confidence intervals were used to display the range of these estimates across the different reference populations.

We also conducted the above analyses using Mikolajczyk et al. 's global reference, which produces birth weight percentiles adaptable to each local population from gestational ages 24 to 41 and is not sex-specific [19]. This distribution differs from those reported above, in that it attempts to identify the true 10th percentile cut-off of the population of interest. This global reference calls for an input of mean birth weight at 40 weeks gestation in the population of interest to produce a distribution; we used 2775 g for Nepal and 2640 g for India.

## Results

A total of 46 reference populations were identified. Ten were excluded from analysis because they did not provide the 10^th^ percentile birth weights, or provided only growth curves. Another ten were excluded, as they were cited less than ten times, leaving a total of 26 reference populations [10]–[13], [16], [20]–[40] (Table S1 in File S1). The gestational age for which these reference populations provided weights ranged from 20–48 weeks. Most reference populations were large; ten studies presented sample sizes of above 100,000, of which seven were above one million. Two studies had sample sizes of less than 2000. North America and Europe were disproportionately represented, with the fewest reference populations from Africa. Most estimated gestational age from date of LMP, although some used ultrasound. All data used to develop the reference populations were from facility-based deliveries. A wide range of inclusion criteria was used. Some included only singletons, others included only live births or those without congenital malformations, and a wide range of maternal characteristics were represented by the different reference populations. Based on the number of times these papers were cited, with older references more likely to be cited than newer ones, some emerged more frequently [10]–[12], [16], [22], [27], [32], [33], [38]. Those from low-income countries and those not published in English were least cited. Five of the references did not provide sex-specific birth-weight-for-gestation curves.

The prevalence of SGA ranged from 10.5% to 72.5% in Nepal, and 12.0% to 78.4% in India, depending on the reference population used to define SGA (Tables 2 and 3). In general, SGA was somewhat higher in India than Nepal. The prevalence of SGA was comparable for males and females except when using reference populations that did not have sex-specific birth-weight-for-gestation curves. In general, females had higher rates of SGA than males using those reference populations. The reference populations from North America and Europe tended to produce higher estimates of SGA relative to those from low-income countries.

**Table 2:** Prevalence of Small-for-Gestational Age (SGA) in Southern Nepal using Reference Populations from Five Regions [17] a.

Reference Region	SGA Definition	All N = 19,966^b^	Male N = 10,237	Female N = 9,727
		% SGA (n)	% SGA (n)	% SGA (n)
**North America**	Alexander [12] 1	52.3 (11, 392)	52.4 (5, 869)	52.1 (5,523)
	Oken [50] 1	62.8 (12,530)	62.8 (6,426)	62.8 (6,104)
	Williams [11] 1	58.6 (11,699)	59.2 (6,057)	58.0 (5,642)
	Lubchenco [16] 1	35.7 (7,124)	35.2 (3,601)	36.2 (3,523)
	Babson [13] c ^,^ 1	57.9 (11,544)	53.0 (5,421)	63.0 (6,123)
	Ott [29] 1	71.9 (14,353)	68.7 (7,031)	75.3 (7,322)
	Brenner [22] c ^,^ 1	45.6 (9,102)	40.5 (4,143)	51.0 (4,959)
	Gruenwald [38] c ^,^ 1	44.5 (8,882)	39.3 (4,026)	50.0 (4,856)
	Freeman (Caucasian) [24] 1	46.6 (9,302)	38.4 (3,933)	55.2 (5,369)
	Freeman (Af. Am) [24] 1	28.0 (5,586)	26.7 (2,727)	29.4 (2,859)
	Zhang [36] 1	62.5 (12,475)	62.8 (6,421)	62.3 (6,054)
	Kramer [10] 1	68.6 (13,694)	68.3 (6,990)	69.0 (6,704)
	Usher [33] ^c.^ 1	58.4 (11,648)	53.6 (5,484)	63.4 (6,164)
**Europe**	Mamelle [25] 1	55.5 (11,066)	56.6 (5,789)	54.3 (5,277)
	Skjaerven [31] 1	72.5 (14,470)	72.9 (7,461)	72.1 (7,009)
	Kloosterman [40] 1	58.7 (11,712)	57.7 (5,906)	59.7 (5,806)
	Parazzini [30] 1	59.1 (11,800)	59.4 (6,076)	58.8 (5,724)
	Thomson [32] 1	56.6 (11,283)	57.9 (5,924)	55.1 (5,359)
	Milner [26] 1	49.4 (9,855)	49.5 (5,063)	49.3 (4,792)
**South America**	Gonzalez [37] c ^, 2^	61.2 (12,209)	56.4 (5,770)	66.2 (6,439)
**Asia**	Bhatia [20] ^3^	12.1 (2,417)	9.9 (1,008)	14.5 (1,409)
	Woo [35] ^2^	50.1 (10,005)	44.9 (4,599)	55.6 (5,406)
	Cheng (Chinese) [23] ^2^	41.4 (8,263)	36.4 (3,726)	46.7 (4,537)
	Cheng (Malay) [23] ^2^	27.1 (5,407)	22.9 (2,340)	31.6 (3,067)
	Cheng (Indian) [23] ^2^	14.4 (2,882)	12.0 (1,231)	17.0 (1,651)
	Nishida [28] 1	49.6 (9,905)	44.5 (4,556)	55.0 (5,349)
	Hong [39] ^2^	56.5 (11,275)	55.9 (5,720)	57.1 (5,555)
**Africa**	Boersma [21] ^3^	10.5 (2,098)	8.6 (878)	12.6 (1,220)
	Verhoeff [34] ^3^	34.7 (6,918)	3,481 (34.0)	35.4 (3,437)

1 High Income, ^2^Middle Income, ^3^Low Income.

a For singleton live births weighed within 72 hours of birth.

b Missing of N = 19,966: gestational age 12, sex 2.

c Reference data are not sex-specific.

**Table 3:** Prevalence of Small-for-Gestational Age (SGA) in South India using Reference Populations from Five Regions [18] a.

Reference Region	SGA Definition	All N = 8,794 b	Male N = 4,504	Female N = 4,290
		% SGA (n)	% SGA (n)	% SGA (n)
**North America**	Alexander [12] 1	61.5 (5,859)	61.8 (3,011)	61.2 (2,848)
	Oken [50] 1	70.0 (6,153)	70.0 (3,148)	70.1 (3,005)
	Williams [11] 1	64.9 (5,706)	65.8 (2,965)	63.9 (2,741)
	Lubchenco [16] 1	40.8 (3,587)	40.6 (1,829)	40.9 (1,758)
	Babson [13] c ^,^ 1	64.8 (5,694)	60.1 (2,705)	69.7 (2,989)
	Ott [29] 1	77.7 (6,831)	74.3 (3,346)	81.2 (3,485)
	Brenner [22] c ^,^ 1	51.9 (4,559)	46.7 (2,103)	57.3 (2,456)
	Gruenwald [38] c ^,^ 1	51.8 (4,553)	46.7 (2,104)	57.1 (2,449)
	Freeman (Caucasian) [24] 1	53.8 (4,734)	45.1 (2,030)	63.0 (2,704)
	Freeman (Af. Am) [24] 1	33.0 (2,896)	32.2 (1,448)	33.8 (1,448)
	Zhang [36] 1	70.0 (6,156)	70.6 (3,177)	69.4 (2,979)
	Kramer [10] 1	75.0 (6,595)	75.2 (3,386)	74.8 (3,209)
	Usher [33] c ^,^ 1	66.1 (5,815)	61.4 (2,763)	71.1 (3,052)
**Europe**	Mamelle [25] 1	62.9 (5,527)	63.8 (2,873)	61.9 (2,654)
	Skjaerven [31] 1	78.4 (6,891)	78.8 (3,547)	78.0 (3,344)
	Kloosterman [40] 1	66.3 (5,827)	65.7 (2,956)	66.9 (2,871)
	Parazzini [30] 1	66.8 (5,781)	66.6 (3,001)	64.8 (2,780)
	Thomson [32] 1	63.1 (5,550)	64.7 (2,911)	61.5 (2,639)
	Milner [26] 1	56.9 (5,001)	57.2 (2,577)	56.5 (2,424)
**South America**	Gonzalez [37] c ^, 2^	68.1 (5,990)	63.7 (2,868)	72.8 (3,122)
**Asia**	Bhatia [20] ^3^	13.5 (1,191)	11.7 (525)	15.5 (666)
	Woo [35] ^2^	56.8 (4,996)	51.5 (2,318)	62.4 (2,678)
	Cheng (Chinese) [23]_2_	46.8 (4,113)	41.9 (1,886)	51.9 (2,227)
	Cheng (Malay) [23] ^2^	30.8 (2,706)	26.7 (1,204)	35.0 (1,502)
	Cheng (Indian) [23] ^2^	15.3 (1,341)	13.1 (589)	17.5 (752)
	Nishida [28] 1	56.5 (4,965)	51.5 (2,321)	61.6 (2,644)
	Hong [39] ^2^	63.0 (5,543)	62.8 (2,828)	63.3 (2,715)
**Africa**	Boersma [21] ^3^	12.0 (1,056)	10.1 (454)	14.0 (602)
	Verhoeff [34] ^3^	39.4 (3,465)	40.0 (1,799)	38.8 (1,666)

1 High Income, ^2^Middle Income, ^3^Low Income.

a For singleton live births weighed within 72 hours of birth.

b Missing of N = 8,794: gestational age 1.

c Reference data are not sex-specific.

**Figure 1:**
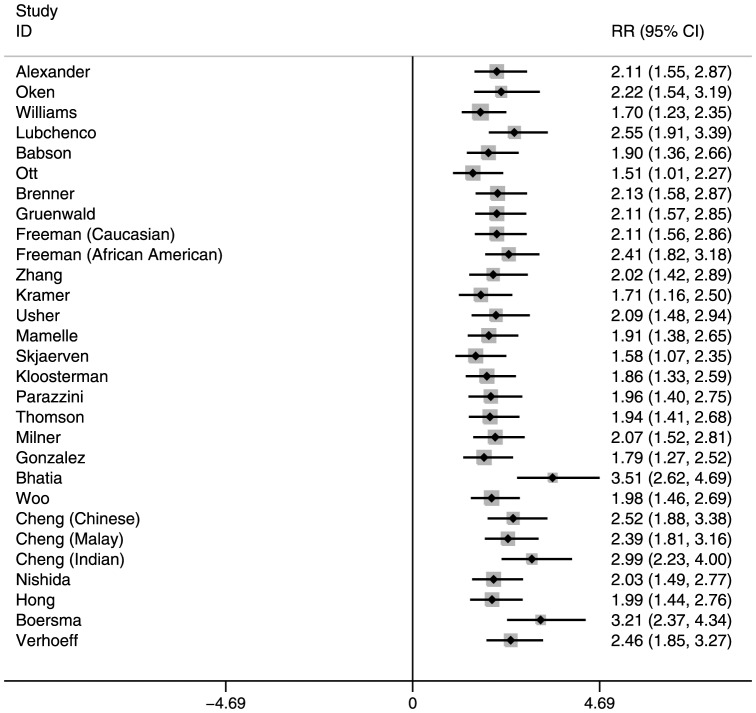
Risk ratios for Term-Small-for-Gestational-Age, Neonatal Mortality: Southern Nepal (reference: Term-Appropriate-for-Gestational-Age).

**Figure 2:**
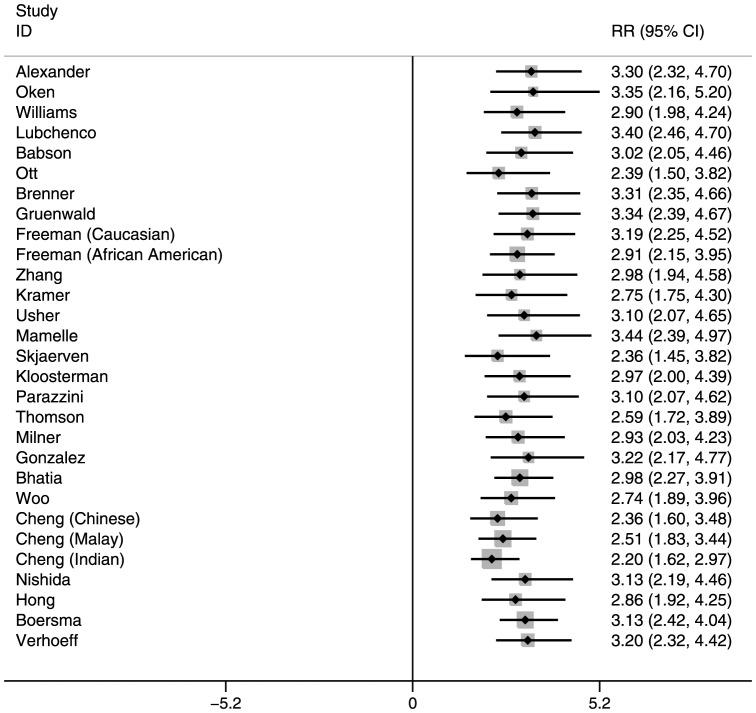
Risk ratios for Preterm-Appropriate-for-Gestational-Age, Neonatal Mortality: Southern Nepal (reference: Term-Appropriate-for-Gestational-Age).

**Figure 3:**
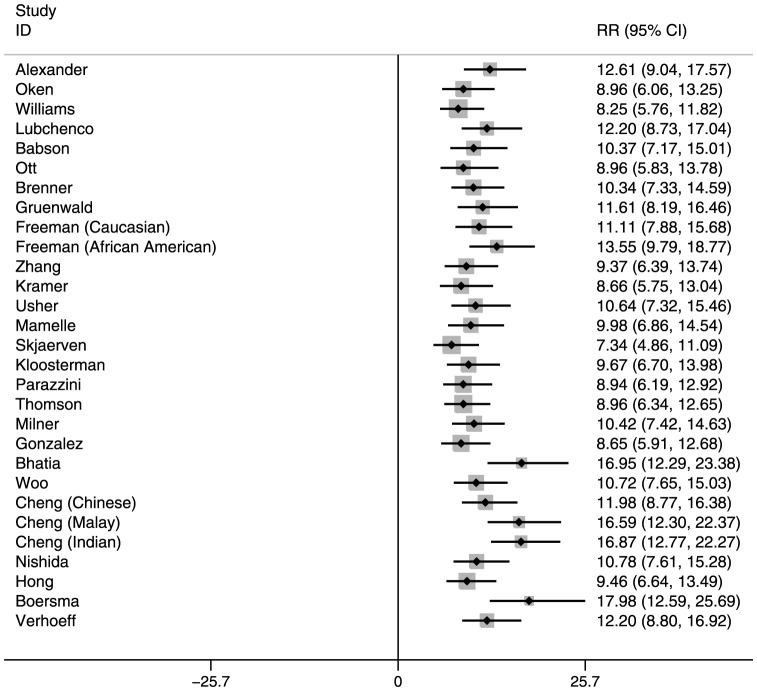
Risk ratios for Preterm-Small-for-Gestational-Age, Neonatal Mortality: Southern Nepal (reference: Term-Appropriate-for-Gestational-Age).

The RR of neonatal mortality among SGA and/or preterm infants also varied by reference population (Tables S2 and S3 in File S1, Figures 1, 2, and 3 for Nepal, and Figures S1, S2, and S3 in File S1 for India). In general, the RRs were highest for reference populations where the prevalence of SGA was lowest. This is because when SGA prevalence is low, very few infants are categorized as SGA, and therefore their mortality risk is high compared with the majority categorized as AGA, resulting in higher RRs. Compared with infants who were term-AGA, term-SGA infants had a significantly higher risk of mortality. Those who were preterm–AGA had a similar increased mortality risk. However, infants who were both preterm and SGA had much higher mortality risk than those who were neither SGA nor preterm. These RRs for preterm-SGA babies ranged from 7.34–17.98 in Nepal and 5.29–11.98 in India, depending on the reference population selected.

The same methodology was used to calculate prevalence and RR as above (i.e. using the 24 week weight cut-off for babies with <24 week gestation and using the 41 week weight cut-off for babies with ≥42 week gestation) with Mikolajczyk et al. 's global reference distribution. This produced a prevalence of 14.5% (male 12.0%, female 17.1%) (fourth lowest, relative to the other reference distributions) for Nepal and 11.0% (male 8.8%, female 13.3%) (lowest) for India. The prevalences were similar when limiting the data to those within the gestational age range that has specific birth-weight cut-offs available (24–41 weeks gestation). In both cases, the term-SGA and preterm-SGA RRs were among the three highest when compared to the other reference distributions, while the preterm-AGA RRs were around the median value (Table S4 in File S1).

## Discussion

A large variety of reference populations have been used to define SGA, making it difficult to pool estimates of SGA prevalence and its mortality consequences using the published literature. The prevalence of SGA and its association with neonatal mortality can vary significantly depending on the choice of reference population. Similar variation has been seen for associations between SGA and developmental outcomes [41]. Reference populations from low-income countries generally produced lower prevalence estimates but higher RRs for neonatal mortality than those from Europe and North America. The much higher prevalence of SGA when using high- versus low-income country reference standards may be attributed to high-income countries producing heavier babies, thereby creating a much higher weight cutoff for SGA than low-income country references. If the birth weight distributions in our Nepal and India datasets were the same as the reference population, the prevalence of SGA should be 10%; these are likely to be the highest risk infants in the distribution. This was approximately what was seen when using Mikolajczyk et al. 's global reference distribution [19], which seeks to create a distribution of birth-weight-for-gestation in the local population. This distribution, which produced prevalences close to 10% for both Nepal and India studies, reported RRs for term- and preterm-SGA that were among the highest compared to other reference distributions.

Some populations (those representing all births in a country over a certain time period) are intended to provide an optimal growth population by considering those below the 10th percentile as exhibiting poor growth. The use of a local reference population may also document progress over time as well as tracking morbidity and mortality risk. Mikolajczyk et al's global reference distribution attempts to identify the lowest 10% within each population. The reference distribution that adapts to the local distribution may successfully identify babies at highest risk in that particular population, but is merely descriptive. It fails to acknowledge that a large percent of the population beyond the lowest 10% also have increased risk of mortality or morbidities when compared to an ideally nourished reference, and does not comment on how the fetus should be growing.

Goldenberg et al examined the issue of variation in reference populations by comparing the weights that defined the 10^th^ percentile cut-off across 13 different reference populations from high-income countries [42]. They found that weights varied across the reference populations from ∼160 g to 820 g depending on weeks of gestation, with the greatest variation at 42 weeks. The reference populations varied by socioeconomic status, the inclusion of multiple births, the proportion of infants who were primiparous, and other factors that could influence the weight cut-offs. However, the authors attribute the most variation in weight cut-offs to the method of gestational age estimation rather than to these other factors. Gardosi et al. have advocated customized birth-weight-for-gestation charts that include maternal weight at first antenatal visit, height, ethnicity, and parity [14]. They showed that the addition of these covariates to the gestational age and sex of the infant reduced misclassification of both small- and large-for-gestational-age (above the 90th percentile in birth weight of a reference population at a specific gestational age) in a population of 4179 women in the United Kingdom. Similar findings were obtained from Australia [43]. While such customized growth charts are valuable for individual diagnosis and clinical use, they are less practical for estimating the prevalence of SGA in populations, especially in low-income settings where certain maternal characteristics may not be collected. In particular, a common reference population, regardless of whether it represents optimal growth, is valuable for comparison of prevalence of SGA and mortality risk across different populations.

Our use of two large, population-based data sets from two different areas of South Asia provided similar results. Hence it is likely these results are generalizable throughout South Asia. By comparing SGA prevalence generated from different reference populations within the same data set, we removed the variability associated with study differences, which would normally be present when comparing SGA prevalence in the published literature.

One limitation of this analysis was the use of dates of LMP by the two studies to estimate gestational age. LMP estimates tend to shift the gestational age distribution to the right [44], [45] and may be associated with substantial misclassification of preterm birth compared to ultrasound-based dating [46]. This misclassification may differentially impact the SGA prevalence; in general, the tendency would be to increase SGA prevalence and decrease relative mortality risk compared with ultrasound. Although most of the reference populations used LMP dates, there is variation in the methods used to determine gestational age (some ultrasound or best obstetric estimate) and LMP dates may be more accurate in certain settings than others (i.e., where literacy is higher). Such variations in gestational age estimation across reference populations likely introduced additional variation in our findings.

In both of these studies, birth weight was measured with reasonable accuracy because they were research studies. Data collectors who were study employees were trained to follow a standard protocol using accurate scales that were calibrated regularly throughout the study. There was likely some inter-observer variability, but we believe this to be a minor source of misclassification. Table S1 in File S1 provides some information on the way in which variables were collected across reference populations, but quality of birth weight measures could have been quite different and these could have added to the variation in prevalence across reference populations.

Another limitation is the range of gestational ages for which weight cut-offs were provided. For the gestational ages lower or higher than the bounds provided by the reference distribution, we used the 10th percentile cut-off of the closest gestational age. By doing so, we may be overestimating SGA prevalence in the highest gestational ages while underestimating SGA prevalence in the lower gestational ages.

It should be noted that our estimates of SGA prevalence and neonatal mortality risk are biased by the exclusion of infants who were missing birth weight or who were weighed beyond 72 hours after delivery. Exclusion of infants who died before being weighed will tend to reduce the prevalence of preterm and SGA since these infants likely died because they had one or both these conditions. However, exclusion of infants weighed after 72 hours of age would likely bias the prevalence of preterm and SGA in the opposite direction, since they would be less likely to be preterm and/or SGA if they survived to 72 hours or beyond. Neither of these biases should impact the estimates of variation by reference population since the comparisons use the same cohort of infants.

Finally, live and stillbirths could have been misclassified in both the trials and reference populations, perhaps more so in these two studies and in low- than high-income reference populations. This misclassification could have altered the estimates of RR but would not alter the comparison of RR across reference populations within each of the trials.

## Conclusion

These results demonstrate the importance of reaching agreement on the appropriate reference population that should be used in future analyses where the primary purpose is to compare SGA prevalence across populations and to estimate global and regional SGA attributable burden. Local fetal growth references may still be useful when considering growth of individual infants in resource limited settings. As has been done with child growth standards [47], The INTERGROWTH-21st Study has collected fetal and neonatal growth measures from healthy women in eight countries, using ultrasound to determine gestational age [48]. A similar activity within the U.S. context is the recently completed NICHD Fetal Growth Study following low risk pregnancies at twelve sites. Whether these combined data will ultimately represent ideal fetal growth curves is not known, but they will produce one common reference population against which **SGA** prevalence and health risks can be assessed. Prior literature will need to be re-evaluated against these new standards, as was done for child growth [49]. These data demonstrate the importance of a common reference population and emphasize the value of the INTERGROWTH-21st Study to be completed in 2014.

## Supporting Information

File S1 **Tables S1–S4 and Figures S1–S3.** Table S1. Comparison of Small-for-Gestational-Age Definitions: Reference Populations. ^*^ LMP  =  Date of last menstrual period; gestational age calculated using the period between date of birth and LMP. Text in italics and gray: population 10th percentile birth weight not available or only given as growth curves. Table S2. Risk ratios for Neonatal Mortality by Preterm and/or Small-for-Gestational-Age: Southern Nepal. Bolded: p<0.05; *Italicized*: 0.05≤p<0.10. ^*^ Preterm defined as gestational age≤37 weeks. ^**^ SGA (small for gestational age) defined as birth weight below the 10^th^ percentile for gestational age. Note: Percentages may not add up to 100.0% due to rounding. Table S3. Risk ratios for Neonatal Mortality by Preterm and/or Small-for-Gestational-Age: South India. ^*^ Preterm defined as gestational age ≤37 weeks. ^**^ SGA (small for gestational age) defined as birth weight below the 10^th^ percentile for gestational age. Note: Percentages may not add up to 100.0% due to rounding. Table S4. Small-for-Gestational-Age prevalence and risk ratio of neonatal mortality, using Mikolajczyk et al. 's global reference birth weight distribution. *Content in the parentheses represent its ranking relative to prevalence/RRs reported for the other reference distributions. Figure S1 in File S1. Risk ratios for Term-Small-for-Gestational-Age, Neonatal Mortality: South India (reference: Term-Appropriate-for-Gestational-Age). Figure S2 in File S1. Risk ratios for Preterm-Appropriate-for-Gestational-Age, Neonatal Mortality: South India (reference: Term-Appropriate-for-Gestational-Age). Figure S3 in File S1. Risk ratios for Preterm-Small-for-Gestational-Age, Neonatal Mortality: South India (reference: Term-Appropriate-for-Gestational-Age).
(PDF)
