# Supplementary material for: Prevalence of Small-for-Gestational-Age and Its Mortality Risk Varies by Choice of Birth-Weight-for-Gestation Reference Population
Source: PLoS One. 2014 Mar 18;9(3):e92074. doi: 10.1371/journal.pone.0092074 (PMC3958448; doi:10.1371/journal.pone.0092074)

Table S1. Comparison of Small-for-Gestational Age (SGA) Definitions: Reference Populations

Region	SGA Definition	Year	Location	Setting or Source	Number of live births	Range of Gestational Age Included	Method of Determining Gestational Age	Racial/Ethnic Characteristics of Reference Population	Other Notes	# Times Cited in Literature	
										Web of Science	Google Scholar
North America	Alexander et al [12]	1991	United States	Nationwide population from 1991 U.S. Live Birth File, National Center for Health Statistics	3,134,8 79	20-44 weeks	LMP*	Not stated	Single births only	497	676
	Oken et al [49]	1999-2000	United States	National Center for Health Statistics 1999 and 2000 Natality Data Sets	6,690,7 17	22-44 weeks	LMP	Data on non-Hispanic whites and non-Hispanic blacks available	Single births only	-	124
	Williams et al [11]	1970-1976	California, United States	Matched birth, death, and fetal death certificates from Center for Health Statistics, California Dept. of Health Services	2,288,8 06	22-48 weeks	LMP	59.2% non-Spanish whites 25.8% Spanish-surname whites 9.9% Black 5.1% other non-white minorities	Single births only	510	549

Region	SGA Definition	Year	Location	Setting or Source	Number of live births	Range of Gestational Age Included	Method of Determining Gestational Age	Racial/Ethnic Characteristics of Reference Population	Other Notes	# Times Cited in Literature	
										Web of Science	Google Scholar
North America	Lubchenco et al [16]	July 1948-January 1961	Denver, Colorado, United States	Admitted to Colorado General Hospital in Denver	5,635	24-42 weeks	LMP	White infants, including Hispanic and non-Hispanic	Mostly low SES; High elevation; Data for babies >36 wks gestation admitted after 1955 excluded due to large numbers	1331	1122
	Babson et al [13]	1959-1966 (except 1964 records misplaced)	Portland, Oregon, United States	All deliveries in two maternity hospitals in Portland, Oregon	39.742	27-44	LMP	White infants	Mostly middle-class; Not sex-specific	135	117
	Ott [29]	1990	St. Louis, Missouri, United States	Computerized discharge data from all live-born infants delivered at St. John's Mercy Medical Ctr between 1990-1991	5,757	20-44 weeks	70% Ultrasound 30% LMP	Not stated	Not sex-specific; Primarily middle- and upper class	78	94

Region	SGA Definition	Year	Location	Setting or Source	Number of live births	Range of Gestational Age Included	Method of Determining Gestational Age	Racial/Ethnic Characteristics of Reference Population	Other Notes	# Times Cited in Literature	
										Web of Science	Google Scholar
North America	Brenner et al [22]	1972-1975 (N. Carolina) 1962-1969 (Cleveland)	Cleveland, Ohio and North Carolina, United States	Abortions at the Memorial Hospital, Univ. of North Carolina at Chapel Hill Live births from University Hospitals of Cleveland, Ohio	430 fetuses 30,772 live births	8-20 weeks (abortions) 21-44 weeks (live births)	LMP	Abortions: 50.8% White 49.2% Nonwhite Live births: 53.3% White 46.7% Nonwhite	Not sex-specific; Birth weights rounded to nearest 10g	715	601
	Gruenwald [38]	1961	United States	Sinai Hospital and the Johns Hopkins University	12,500	28-43	LMP and obstetrician confirmation	Not stated	Multiple births and malformations that affected birth weight excluded; Not sex-specific	286	307
	Freeman [24]	1959-1965 (Caucasian) 1965-1966 (African American)	Atlanta, Georgia, United States	Hospital records from deliveries at the Grady Memorial Hospital (large metropolitan setting)	7,547 Caucasian 9,800 African American	28-46 weeks	LMP	See Number of live births	Population classified as 'indigent'; Stratified by race: Caucasian and African American; Excluded twins and stillborns	-	96

Region	SGA Definition	Year	Location	Setting or Source	Number of live births	Range of Gestational Age Included	Method of Determining Gestational Age	Racial/Ethnic Characteristics of Reference Population	Other Notes	# Times Cited in Literature	
										Web of Science	Google Scholar
North America	Zhang and Bowes [36]	1989	United States	Computer certifications of live births in 1989, from National Center for Health Statistics	4,040,958	25-42	LMP plus clinical estimate in outliers	Only white or black (insufficient number of births of other races) Mixed race births assigned to other parent's race if one parent was white	Singleton births only	149	180
	Kramer et al [10]	1994-1996	Canada	Nationally linked live birth-infant death file for infants born in all provinces except Ontario	676,605	22-43 weeks	Ultrasound	Unknown: no information on racial origin contained in Canadian birth certificates	Single births only	160	216
	Usher & McLean [33]	mostly 1959, premature infants added to study until 1963	Montreal, Canada	Deliveries in the Royal Victoria Hospital	300	25-44	LMP	Caucasian infants only from "varying national origins"	Single births only;<br>SES varied;<br>Not sex-specific	973	754

Region	SGA Definition	Year	Location	Setting or Source	Number of live births	Range of Gestational Age Included	Method of Determining Gestational Age	Racial/Ethnic Characteristics of Reference Population	Other Notes	# Times Cited in Literature	
										Web of Science	Google Scholar
Europe	Mamelle et al [25]	1984-1988	France	22 Maternity hospitals in various French regions	97,222	28-44 weeks	Ultrasound	20% of mothers of non-French origin		-	23
	Skjaerven [31]	1967-1998	Norway	Medical Birth Registry	1,709,311	20-44	LMP	Not stated	Excludes stillbirths, malformations, and C-sections	107	150
	Kloosterman [40]	1948-1957 1931-1965 Published 1969	Amsterdam (Holland)	University clinic	Says 80,000 but table shows 20,696	20-46	LMP	Not stated	Sex-specific	-	40
Europe	Parazzini et al [30]	1984-1985 Published 1991	Italy	Italian Central Institute of Statistics	1,200,000 1,169,564 based on Table I	28-43	Not stated	Not stated. Appears to be all births in Italy during this time period	Sex-specific	-	25
	Thomson & Billewicz [32]	1948-1964	England Aberdeen, Scotland	>80% births took place in one teaching maternity hospital	46,703	32-42	LMP	~95% of inhabitants of Aberdeen born in Scotland	Single births only; Sex-specific	-	473
	Milner & Richards [26]	1967-1971	England and Wales	Deliveries in National Health Service (NHS) hospitals	271,519	28-44	LMP	Not stated	Single births only; Sex-specific	126	88

Region	SGA Definition	Year	Location	Setting or Source	Number of live births	Range of Gestational Age Included	Method of Determining Gestational Age	Racial/Ethnic Characteristics of Reference Population	Other Notes	# Times Cited in Literature	
										Web of Science	Google Scholar
South America	Gonzalez et al [37]	1993-2000	Chile	National database of deliveries	2,049,446	22-42	LMP and ultrasound	Not stated	Singletons only; Sex-specific data in supplement (unable to locate)	25	33
Asia	Bhatia et al [20]	1976-1980	India	Population from Varanasi and Delhi (Methods section does not clearly state source of data)	5,321	26-44	LMP	Not stated	Single live births only; Excludes congenital malformations and mothers with diabetes, heart disease, or hydramnios; Not sex-specific	-	12
	Woo et al [35]	1982-1984	Hong Kong	Live births in 2 maternity teaching hospitals in Hong Kong	15,815	28-42	LMP	99.8% of Chinese descent	Single births only; Mostly middle and lower social classes; Not sex-specific	9	14
	Cheng et al [23]	1968-1970	Singapore	Deliveries at the University Unit in Kandang Kerbau Hospital	4,506 Chinese 3,722 Malay 2,798 Indian	34-42	LMP	See Number of Live Births (According to hospital records, approx. 75% of births Chinese, 15% Malay, 10% Indian)	Stratified by race; Stillbirths, gross congenital malformation, multiple births excluded; Not sex-specific	-	15
	Nishida et al [28]	1985	Japan	Data from 37 major medical centers throughout Japan	5,608	24-44	LMP	Not stated	Single births only; Not sex-specific	0	28

Region	SGA Definition	Year	Location	Setting or Source	Number of live births	Range of Gestational Age Included	Method of Determining Gestational Age	Racial/Ethnic Characteristics of Reference Population	Other Notes	# Times Cited in Literature	
										Web of Science	Google Scholar
	Hong et al [39]	1999	Korea	Vital statistics linked National Infant Mortality Survey from 4513 medical institutions	609,643 singletons 9805 twins	23-42	LMP	Not stated	Includes singletons and twins; Excluded triplets and higher order births due to small numbers	1	129
Africa	Boersma & Mbise [21]	Sept. 1975-March 1976	Dar es Salaam, Tanzania	Two public hospitals in Dar es Salaam: Muhimbili and Ocean Road	entire study: 16,532	28-42 (data given for every two weeks)	From Lubchenco 1970	~90% of deliveries in the city at these two hospitals and states 'nearly all are Bantu African Americanes' May have small numbers of Asian descent	Data in tables are not sex-specific; Singletons only; Excludes congenital malformations and maternal chronic disease	-	19
	Verhoeff et al [34]	March 1993-June 1994 (Pub 2001)	Southern Malawi	Two hospitals in rural Chikwawa District	1423	28-43	Postnatal exam using Ballard method	Not stated	Malaria-endemic area; Singleton births; Sex-specific	19	27

[*] LMP = Date of last menstrual period; gestational age calculated using the period between date of birth and LMP

Text in italics and gray: population 10th percentile birth weight not available or only given as growth curves

Table S2. Risk ratios for Neonatal Mortality by Preterm and/or Small-for-gestational-age: Southern Nepal[17]

Reference Region	SGA Definition	RR (95% CI)<br>n (%) of N=19,966			
		Term-AGA (reference)	Term-SGA	Preterm-AGA	Preterm*-SGA**
North America	Alexander [12]	1.00<br>7504 (34.4%)	**2.11 (1.55 – 2.87)**<br>10,418 (47.8%)	**3.30 (2.32 - 4.70)**<br>2906 (13.3%)	**12.61 (9.04 – 17.57)**<br>970 (4.5%)
	Oken[49]	1.00<br>5468 (26.9%)	**2.22 (1.54 - 3.19)**<br>11,127 (55.8%)	**3.35 (2.16 - 5.20)**<br>2056 (10.3%)	**8.96 (6.06 - 13.25)**<br>1403 (7.0%)
	Williams [11]	1.00<br>5855 (29.3%)	**1.70 (1.23 - 2.35)**<br>10,635 (53.3%)	**2.90 (1.98 - 4.24)**<br>2400 (12.0%)	**8.25 (5.76 - 11.82)**<br>1064 (5.3%)
	Lubchenco [16]	1.00<br>10,009 (50.2%)	**2.55 (1.91 - 3.39)**<br>6481 (32.5%)	**3.40 (2.46 - 4.70)**<br>2821 (14.1%)	**12.20 (8.73 - 17.04)**<br>643 (3.2%)
	Babson[13†]	1.00<br>5857 (29.4%)	**1.90 (1.36 - 2.66)**<br>10,633 (53.3%)	**3.02 (2.05 - 4.46)**<br>2553 (12.8%)	**10.37 (7.17 - 15.01)**<br>911 (4.6%)
	Ott [29]	1.00<br>3212 (16.1%)	**1.51 (1.01 - 2.27)**<br>13,278 (66.5%)	**2.39 (1.50 - 3.82)**<br>2389 (12.0%)	**8.96 (5.83 - 13.78)**<br>1075 (5.4%)
	Brenner[22 †]	1.00<br>8179 (41.0%)	**2.13 (1.58 - 2.87)**<br>8311 (41.7%)	**3.31 (2.35 - 4.66)**<br>2673 (13.4%)	**10.34 (7.33 - 14.59)**<br>791 (4.0%)
	Gruenwald [38†]	1.00<br>8251 (41.4%)	**2.11 (1.57 - 2.85)**<br>8239 (41.3%)	**3.34 (2.39 - 4.67)**<br>2821 (14.1%)	**11.61 (8.19 - 16.46)**<br>643 (3.2%)
	Freeman (Caucasian) [24]	1.00<br>7951 (39.8%)	**2.11 (1.56 - 2.86)**<br>8539 (42.8%)	**3.19 (2.25 - 4.52)**<br>2701 (13.5%)	**11.11 (7.88 - 15.68)**<br>763 (3.8%)
	Freeman (Af. Am)	1.00<br>11,376 (57.0%)	**2.41 (1.82 - 3.18)**<br>5122 (25.7%)	**2.91 (2.15 - 3.95)**<br>2992 (15.0%)	**13.55 (9.79 - 18.77)**<br>464 (2.3%)
	Zhang [36]	1.00<br>5728 (26.5%)	**2.02 (1.42 - 2.89)**<br>11,212 (56.2%)	**2.98 (1.94 - 4.58)**<br>2201 (11.0%)	**9.37 (6.39 - 13.74)**<br>1263 (6.3%)
	Kramer [10]	1.00<br>4004 (20.1%)	**1.71 (1.16 - 2.50)**<br>12,486 (62.6%)	**2.75 (1.75 - 4.30)**<br>2256 (11.3%)	**8.66 (5.75 - 13.04)**<br>1208 (6.1%)
	Usher [33†]	1.00<br>5864 (29.4%)	**2.09 (1.48 - 2.94)**<br>10,626 (53.3%)	**3.10 (2.07 - 4.65)**<br>2442 (12.2%)	**10.64 (7.32 - 15.46)**<br>1022 (5.1%)
Europe	Mamelle [25]	1.00<br>6204 (31.1%)	**1.91 (1.38 - 2.65)**<br>10,286 (51.6%)	**3.44 (2.39 - 4.97)**<br>2684 (13.5%)	**9.98 (6.86 - 14.54)**<br>780 (3.9%)
	Skjaerven [31]	1.00<br>3558 (17.8%)	**1.58 (1.07 - 2.35)**<br>12,932 (64.8%)	**2.36 (1.45 - 3.82)**<br>1926 (9.7%)	**7.34 (4.86 - 11.09)**<br>1538 (7.7%)
	Kloosterman [40]	1.00<br>5767 (28.9%)	**1.86 (1.33 - 2.59)**<br>10,723 (53.7%)	**2.97 (2.00 - 4.39)**<br>2475 (12.4%)	**9.67 (6.70 - 13.98)**<br>989 (5.0%)
	Parazzini [30]	1.00<br>5864 (29.4%)	**1.96 (1.40 - 2.75)**<br>10,626 (53.3%)	**3.10 (2.07 - 4.62)**<br>2290 (11.5%)	**8.94 (6.19 - 12.92)**<br>1174 (5.9%)
	Thomson [32]	1.00<br>6476 (32.5%)	**1.94 (1.41 - 2.68)**<br>10,014 (50.2%)	**2.59 (1.72 - 3.89)**<br>2195 (11.0%)	**8.96 (6.34 - 12.65)**<br>1269 (6.4%)
	Milner [26]	1.00<br>7565 (37.9%)	**2.07 (1.52 - 2.81)**<br>8925 (44.7%)	**2.93 (2.03 - 4.23)**<br>2534 (12.7%)	**10.42 (7.42 - 14.63)**<br>930 (4.7%)
South America	Gonzalez[37†]	1.00<br>5296 (26.5%)	**1.79 (1.27 - 2.52)**<br>11,194 (56.1%)	**3.22 (2.17 - 4.77)**<br>2449 (12.3%)	**8.65 (5.91 - 12.68)**<br>1015 (5.1%)
Asia	Bhatia [20]	1.00<br>14,362 (72.0%)	**3.51 (2.62 - 4.69)**<br>2128 (10.7%)	**2.98 (2.27 - 3.91)**<br>3175 (15.9%)	**16.95 (12.29 - 23.38)**<br>289 (1.5%)
	Woo [35]	1.00<br>7392 (37.0%)	**1.98 (1.46 - 2.69)**<br>9098 (45.6%)	**2.74 (1.89 - 3.96)**<br>2557 (12.8%)	**10.72 (7.65 - 15.03)**<br>907 (4.6%)
	Cheng (Chinese) [23]	1.00<br>9257 (46.4%)	**2.52 (1.88 - 3.38)**<br>7233 (36.3%)	**2.36 (1.60 - 3.48)**<br>2434 (12.2%)	**11.98 (8.77 - 16.38)**<br>1030 (5.2%)
	Cheng (Malay)	1.00<br>11,557 (57.9%)	**2.39 (1.81 - 3.16)**<br>4933 (24.7%)	**2.51 (1.83 - 3.44)**<br>2990 (15.0%)	**16.59 (12.30 - 22.37)**<br>474 (2.4%)
	Cheng (Indian)	1.00<br>14,047 (70.4%)	**2.99 (2.23 - 4.00)**<br>2443 (12.2%)	**2.20 (1.62 - 2.97)**<br>3025 (15.2%)	**16.87 (12.77 - 22.27)**<br>439 (2.2%)
	Nishida [28]	1.00<br>7393 (37.1%)	**2.03 (1.49 - 2.77)**<br>9097 (45.6%)	**3.13 (2.19 - 4.46)**<br>2656 (13.3%)	**10.78 (7.61 - 15.28)**<br>808 (4.1%)

Reference Region	SGA Definition	RR (95% CI)<br>n (%) of N=19,966			
		Term-AGA (reference)	Term-SGA	Preterm-AGA	Preterm[*]-SGA[**]
	Hong [39]	1.00<br>6359 (31.9%)	**1.99 (1.44 - 2.76)**<br>10,131 (50.8%)	**2.86 (1.92 - 4.25)**<br>2320 (11.6%)	**9.46 (6.64 - 13.49)**<br>1144 (5.7%)
**Africa**	Boersma [21]	1.00<br>14,580 (73.1%)	**3.21 (2.37 - 4.34)**<br>1910 (9.6%)	**3.13 (2.42 - 4.04)**<br>3276 (16.4%)	**17.98 (12.59 - 25.69)**<br>188 (0.9%)
	Verhoeff [34]	1.00<br>10,216 (51.2%)	**2.46 (1.85 - 3.27)**<br>6274 (31.4%)	**3.20 (2.32 - 4.42)**<br>2820 (14.1%)	**12.20 (8.80 - 16.92)**<br>644 (3.2%)

Bolded: $p < 0.05$; *Italicized*: $0.05 \le p < 0.10$

[*] Preterm defined as gestational age ≤ 37 weeks

[**] SGA (small for gestational age) defined as birth weight below the 10th percentile for gestational age

Note: Percentages may not add up to 100.0% due to rounding.

Table S3. Risk ratios for Neonatal Mortality by Preterm and/or Small-for-gestational-age: South India[18]

Reference Region	SGA Definition	RR (95% CI)<br>n (%) of N=8794			
		Term-AGA (reference)	Term-SGA	Preterm-AGA	Preterm*-SGA**
North America	Alexander [12]	1.00<br>2705 (28.4%)	2.04 (1.30 - 3.21)<br>5589 (58.7%)	2.79 (1.57 - 4.96)<br>965 (10.13%)	8.28 (4.57 – 15.00)<br>270 (2.8%)
	Oken[49]	1.00<br>1878 (21.4%)	1.70 (1.04 - 2.77)<br>5748 (65.4%)	2.46 (1.31 - 4.62)<br>762 (8.7%)	4.87 (2.63 - 9.05)<br>405 (4.6%)
	Williams [11]	1.00<br>2221 (25.3%)	1.79 (1.13 - 2.84)<br>5403 (61.5%)	2.68 (1.50 - 4.78)<br>867 (9.9%)	5.66 (3.04 - 10.54)<br>303 (3.5%)
	Lubchenco [16]	1.00<br>4222 (48.0%)	1.94 (1.35 - 2.80)<br>3402 (38.7%)	2.33 (1.44 - 3.77)<br>985 (11.2%)	7.44 (4.23 - 13.08)<br>185 (2.1%)
	Babson[13†]	1.00<br>2197 (25.0%)	1.87 (1.17 - 2.99)<br>5427 (61.7%)	2.32 (1.26 - 4.25)<br>903 (10.3%)	7.83 (4.30 - 14.26)<br>267 (3.0%)
	Ott [29]	1.00<br>1096 (12.5%)	2.03 (1.03 - 4.00)<br>6528 (74.2%)	2.53 (1.14 - 5.59)<br>867 (9.9%)	8.83 (4.11 - 18.98)<br>303 (3.5%)
	Brenner[22 †]	1.00<br>3295 (37.%)	1.54 (1.05 - 2.26)<br>4329 (49.2%)	1.98 (1.18 - 3.32)<br>940 (10.7%)	6.61 (3.84 - 11.37)<br>230 (2.6%)
	Gruenwald [38†]	1.00<br>3265 (37.1%)	1.52 (1.04 - 2.22)<br>4359 (49.6%)	2.06 (1.24 - 3.40)<br>976 (11.1%)	6.90 (3.93 - 12.13)<br>194 (2.2%)
	Freeman (Caucasian) [24]	1.00<br>3109 (35.4%)	1.39 (0.95 - 2.04)<br>4515 (51.4%)	2.09 (1.27 - 3.44)<br>951 (10.8%)	5.46 (3.06 - 9.74)<br>219 (2.5%)
	Freeman (Af. Am)	1.00<br>35.4%	2.10 (1.46 - 3.00)<br>2752 (31.3%)	2.48 (1.58 - 3.90)<br>1018 (11.6%)	6.89 (3.68 - 12.90)<br>144 (1.6%)
	Zhang [36]	1.00<br>1839 (20.9%)	2.03 (1.20 - 3.42)<br>5785 (65.8%)	2.73 (1.41 - 5.28)<br>799 (9.1%)	6.50 (3.43 - 12.34)<br>371 (4.2%)
	Kramer [10]	1.00<br>1384 (15.7%)	2.39 (1.26 - 4.56)<br>6240 (71.0%)	3.22 (1.51 - 6.90)<br>815 (9.3%)	8.18 (3.89 - 17.22)<br>355 (4.0%)
	Usher [33†]	1.00<br>2100 (23.9%)	1.49 (0.95 - 2.32)<br>5524 (62.8%)	1.89 (1.04 - 3.43)<br>879 (10.0%)	6.31 (3.56 - 11.19)<br>291 (3.3%)
Europe	Mamelle [25]	1.00<br>2311 (26.3%)	1.70 (1.09 - 2.66)<br>5313 (60.4%)	2.62 (1.51 - 4.54)<br>956 (10.9%)	6.30 (3.31 - 11.99)<br>214 (2.4%)
	Skjaerven [31]	1.00<br>1178 (13.4%)	3.41 (1.50 - 7.73)<br>6446 (73.3%)	4.33 (1.70 - 11.01)<br>725 (8.3%)	10.58 (4.35 - 25.71)<br>445 (5.1%)
	Kloosterman [40]	1.00<br>2073 (23.6%)	1.72 (1.08 - 2.76)<br>5551 (63.1%)	2.76 (1.55 - 4.90)<br>894 (10.2%)	5.36 (2.80 - 10.28)<br>276 (3.1%)
	Parazzini [30]	1.00<br>2180 (24.8%)	1.75 (1.10 - 2.77)<br>5444 (61.9%)	2.62 (1.46 - 4.70)<br>833 (9.5%)	5.29 (2.87 - 9.76)<br>337 (3.8%)
	Thomson [32]	1.00<br>2451 (27.9%)	1.85 (1.19 - 2.90)<br>5173 (58.8%)	2.45 (1.35 - 4.44)<br>793 (9.0%)	5.69 (3.20 - 10.11)<br>377 (4.3%)
	Milner [26]	1.00<br>2894 (32.9%)	1.72 (1.14 - 2.58)<br>4730 (53.8%)	1.97 (1.12 - 3.47)<br>899 (10.2%)	7.23 (4.22 - 12.41)<br>271 (3.1%)
South America	Gonzalez[37†]	1.00<br>1919 (21.8%)	2.14 (1.27 - 3.62)<br>5705 (64.9%)	2.84 (1.49 - 5.42)<br>885 (10.1%)	7.99 (4.16 - 15.36)<br>285 (3.2%)
Asia	Bhatia [20]	1.00<br>6519 (74.1%)	2.59 (1.76 - 3.81)<br>1105 (12.6%)	2.20 (1.46 - 3.33)<br>1084 (12.3%)	9.24 (4.97 - 17.20)<br>86 (1.0%)
	Woo [35]	1.00<br>2901 (33.0%)	1.65 (1.10 - 2.47)<br>4723 (53.7%)	1.92 (1.09 - 3.37)<br>897 (10.2%)	6.97 (4.08 - 11.92)<br>273 (3.1%)
	Cheng (Chinese) [23]	1.00<br>3822 (43.5)	1.82 (1.25 - 2.64)<br>3802 (43.2%)	1.69 (0.96 - 3.00)<br>859 (9.8%)	7.02 (4.31 - 11.44)<br>311 (3.5%)
	Cheng (Malay)	1.00	2.27 (1.58 - 3.24)	1.89 (1.15 - 3.11)	11.98 (7.30 - 19.65)

		5064 (57.6%)	2560 (29.1%)	1024 (11.7%)	146 (1.7%)
	Cheng (Indian)	1.00<br>6424 (73.0%)	2.64 (1.81 - 3.86)<br>1200 (13.7%)	1.58 (0.97 - 2.57)<br>1029 (11.7%)	11.53 (7.27 - 18.29)<br>141 (1.6%)
	Nishida [28]	1.00<br>2886 (32.8%)	1.57 (1.05 - 2.34)<br>4738 (53.9%)	1.95 (1.13 - 3.35)<br>943 (10.7%)	7.32 (4.23 - 12.66)<br>227 (2.6%)
	Hong [39]	1.00<br>2412 (27.4%)	1.81 (1.16 - 2.83)<br>5212 (59.3%)	2.39 (1.33 - 4.31)<br>839 (9.5%)	6.07 (3.39 - 10.86)<br>331 (3.8%)
**Africa**	Boersma [21]	1.00<br>6614 (75.2%)	2.54 (1.71 - 3.78)<br>1010 (11.5%)	2.28 (1.54 - 3.40)<br>1124 (12.8%)	11.84 (5.80 - 24.18)<br>46 (0.5%)
	Verhoeff [34]	1.00<br>4352 (49.5%)	2.08 (1.44 - 3.00)<br>3272 (37.2%)	2.23 (1.36 - 3.66)<br>977 (11.1%)	8.33 (4.87 - 14.26)<br>193 (2.2%)

* Preterm defined as gestational age ≤37 weeks

** SGA (small-for-gestational-age) defined as birth weight below the 10th percentile for gestational age

Note: Percentages may not add up to 100.0% due to rounding

Table S4: Small-for-gestational-age prevalence and risk ratios of neonatal mortality, using Mikolajczyk et al.'s global reference birth weight distribution

		Prevalence	RR (95% CI): Term-SGA	RR (95% CI): Preterm-AGA	RR (95% CI): Preterm-SGA
**Nepal**	**All babies**	14.48% (4th lowest)	3.06 (2.32-4.03) (3rd highest)	3.23 (2.50-4.16) (6th highest)	24.92 (18.61-33.36) (highest)
	**Limited to babies 24-41 weeks gestation**	13.31% (3rd lowest)	3.04 (2.26-4.10) (3rd highest)	3.09 (2.38-4.01) (13th highest	23.84 (17.72-32.09) (highest)
**India**	**All babies**	10.99% (lowest)	3.13 (2.13-4.60) (2nd highest)	2.34 (1.56-3.50) (14th highest)	17.67 (9.77-31.96) (highest)
	**Limited to babies 24-41 weeks gestation**	9.78% (lowest)	3.40 (2.20-5.25) (2nd highest)	2.48 (1.64-3.76) (11th highest)	18.77 (10.29-34.25) (highest)

*Content in the parentheses represent its ranking relative to prevalence / RRs reported for the other reference distributions.

Figure S1. Risk ratios for Term-Small-for-gestational-age, Neonatal Mortality: South India (reference: Term-Appropriate-for-gestational-age)

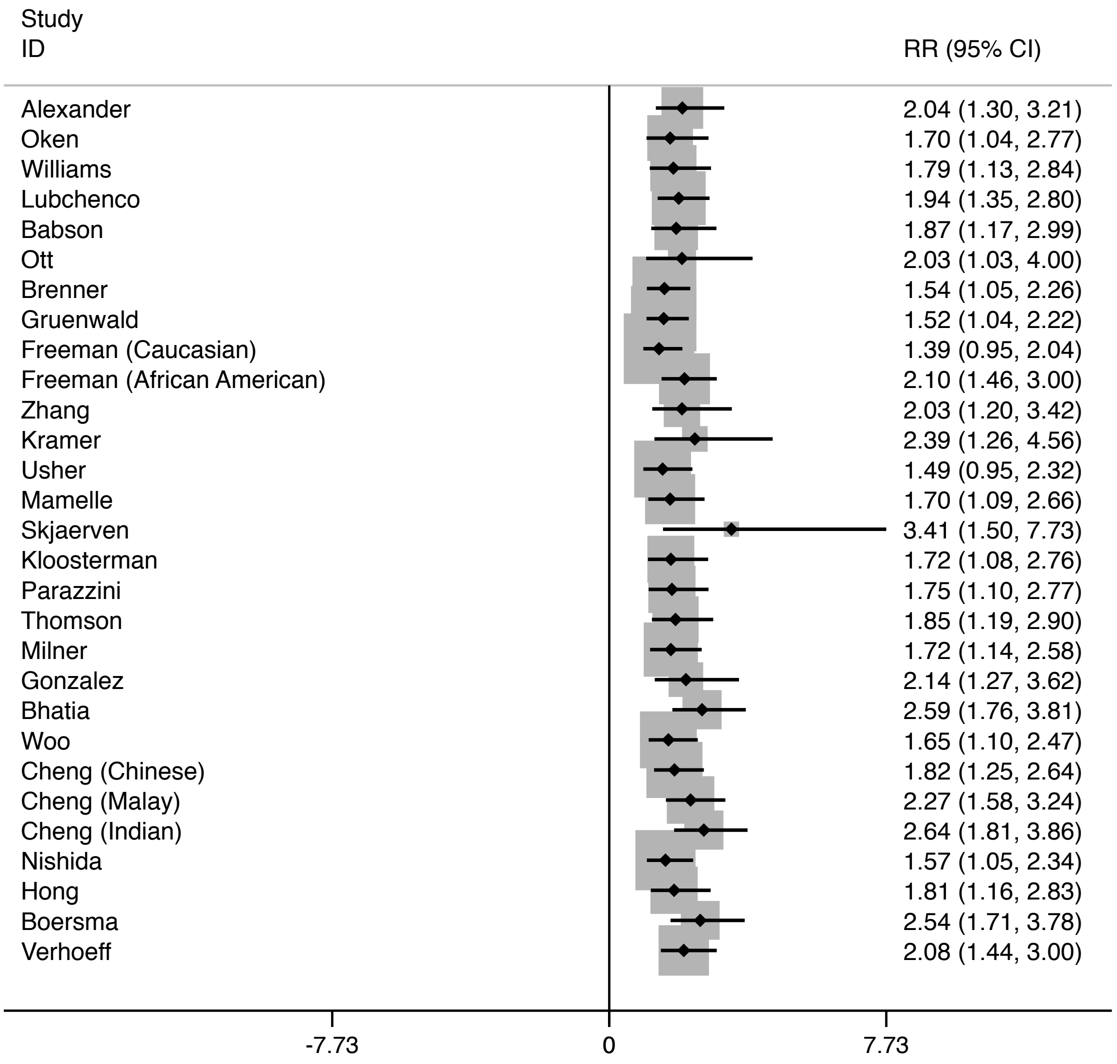

Figure S2. Risk ratios for Preterm-Appropriate-for-gestational-age, Neonatal Mortality: South India (reference: Term-Appropriate-for-gestational-age)

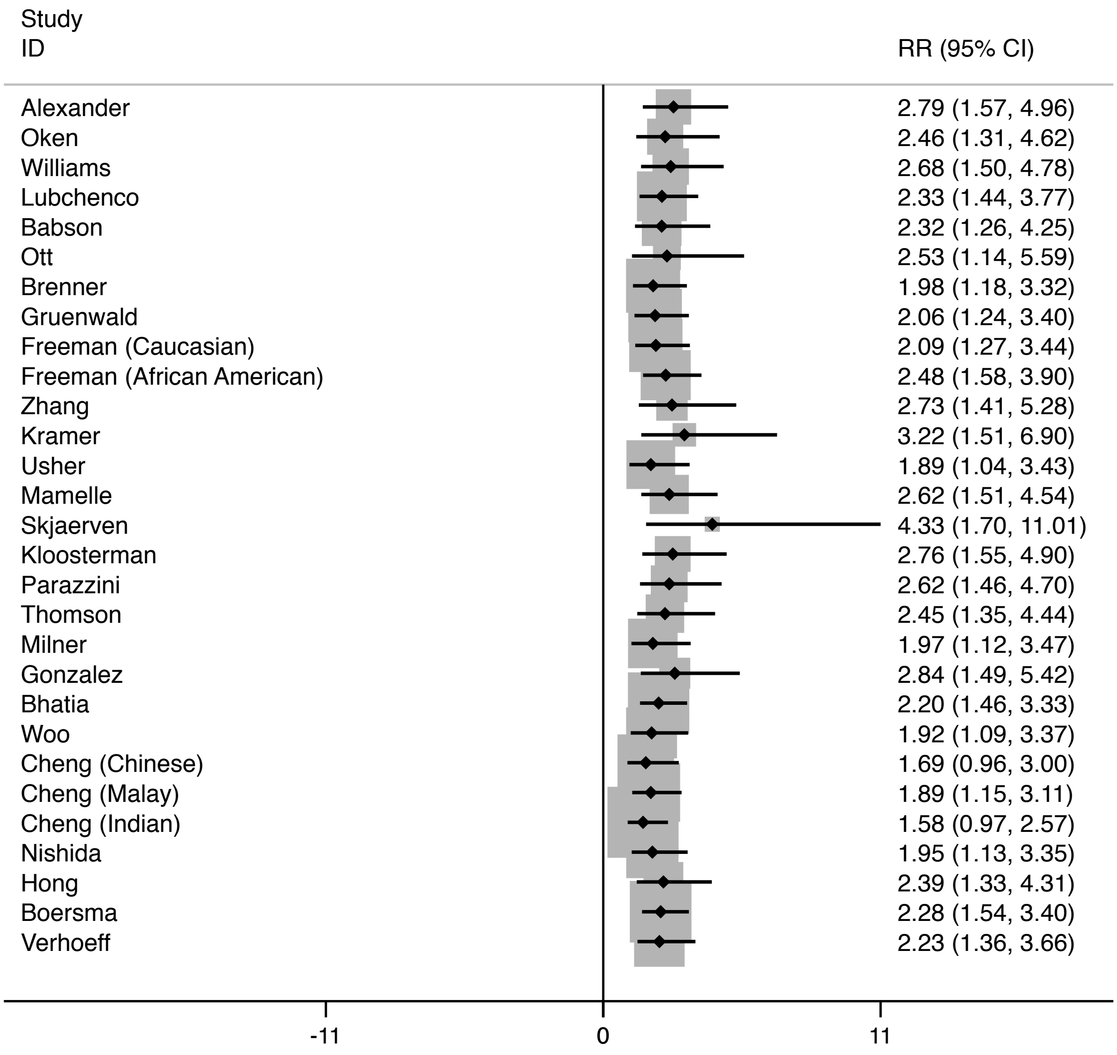

Figure S3. Risk ratios for Preterm-Small-for-gestational-age, Neonatal Mortality: South India (reference: Term-Appropriate-for-gestational-age)

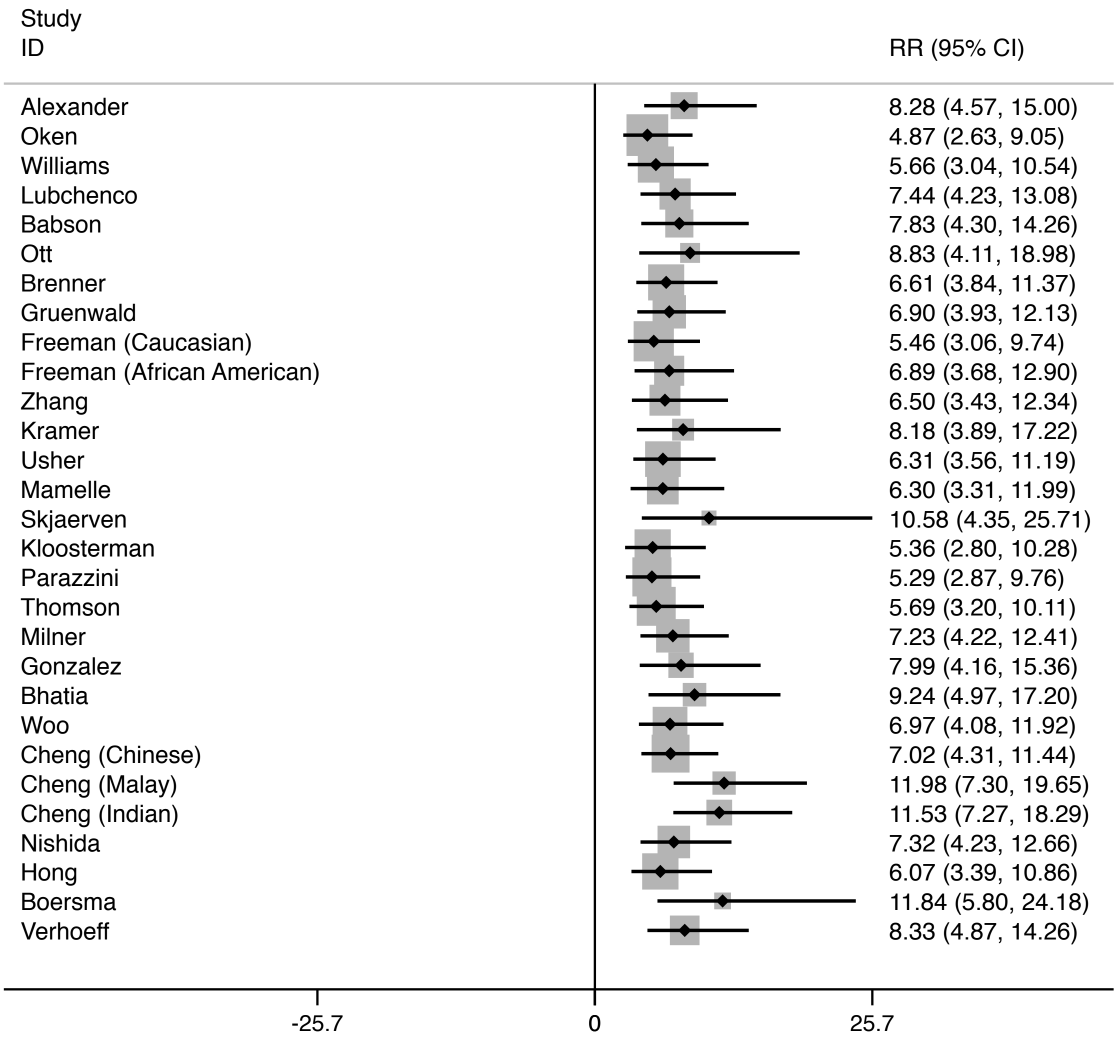

Supplement: File S1 — Tables S1–S4 and Figures S1–S3. Table S1. Comparison of Small-for-Gestational-Age Definitions: Reference Populations. * LMP = Date of last menstrual period; gestational age calculated using the period between date of birth and LMP. Text in italics and gray: population 10th percentile birth weight not available or only given as growth curves. Table S2. Risk ratios for Neonatal Mortality by Preterm and/or Small-for-Gestational-Age: Southern Nepal. Bolded: p<0.05; Italicized: 0.05≤p<0.10. * Preterm defined as gestational age≤37 weeks. ** SGA (small for gestational age) defined as birth weight below the 10th percentile for gestational age. Note: Percentages may not add up to 100.0% due to rounding. Table S3. Risk ratios for Neonatal Mortality by Preterm and/or Small-for-Gestational-Age: South India. * Preterm defined as gestational age ≤37 weeks. ** SGA (small for gestational age) defined as birth weight below the 10th percentile for gestational age. Note: Percentages may not add up to 100.0% due to rounding. Table S4. Small-for-Gestational-Age prevalence and risk ratio of neonatal mortality, using Mikolajczyk et al. 's global reference birth weight distribution. *Content in the parentheses represent its ranking relative to prevalence/RRs reported for the other reference distributions. Figure S1 in File S1. Risk ratios for Term-Small-for-Gestational-Age, Neonatal Mortality: South India (reference: Term-Appropriate-for-Gestational-Age). Figure S2 in File S1. Risk ratios for Preterm-Appropriate-for-Gestational-Age, Neonatal Mortality: South India (reference: Term-Appropriate-for-Gestational-Age). Figure S3 in File S1. Risk ratios for Preterm-Small-for-Gestational-Age, Neonatal Mortality: South India (reference: Term-Appropriate-for-Gestational-Age). (PDF) [file pone.0092074.s001.pdf]
